# Proposed modifications of supraclavicular lymph node metastasis in the esophageal squamous cell carcinoma staging system for improved survival stratification

**DOI:** 10.18632/oncotarget.14892

**Published:** 2017-01-29

**Authors:** Yuzhen Zheng, Zhen Wang, Feng Wang, Qingyuan Huang, Shuoyan Liu

**Affiliations:** ^1^ Department of Thoracic Oncology, Fujian Cancer Hospital & Fujian Medical University Cancer Hospital, Fuzhou, Fujian, P.R. China; ^2^ Fujian Provincial Key Laboratory of Tumor Biotherapy, Fuzhou, Fujian, P.R. China; ^3^ Department of Thoracic Surgery, Shanghai Jiao Tong University Affiliated Chest Hospital, Shanghai, P.R. China

**Keywords:** esophageal cancer, supraclavicular lymph nodes, prognosis

## Abstract

The present study aims to investigate the clinical implication of supraclavicular lymph nodes (SCLNs) in thoracic esophageal squamous cell carcinoma (ESCC). A total of 1156 ESCC patients who underwent three-field lymphadenectomy with node metastasis were analyzed retrospectively. SCLNs were defined as regional nodes in the current system or as distant nodes in the modified system. Survival was analyzed using the Kaplan-Meier method, and values were compared using the log-rank test. Multivariate analysis was performed using the Cox proportional hazard model. The Akaike information criterion (AIC) and the concordance index (c-index) were applied to compare the two prognostic systems. Among 1156 patients, 183 (15.8%) patients were diagnosed with SCLN metastasis. Higher rate of SCLN metastasis was associated with upper tumor location, metastasis involving seven or more nodes, and positive recurrent laryngeal nerve node status. The current staging system was unable to stratify overall survival well in patients with N2, N3, and M1 status using a univariate analysis. In both the current staging system and the modified version, age, gender, pathological T status, and nodal status were independent prognostic factors in a multivariate analysis. The AIC value for the modified version was smaller than that for the current staging system; the c-index value for the modified version was larger than that for the current staging system. Based on the data from our single center, SCLNs should be reclassified as regional lymph nodes in thoracic ESCC for better stratification of overall survival.

## INTRODUCTION

Esophageal cancer is the eighth most common cancer in the world [1], and lymph node metastasis is one of the most important prognostic factors [2, 3]. In the 7^th^ Union for International Cancer Control (UICC) TNM classification[4] and in the 7^th^ American Joint Committee On Cancer (AJCC) Staging Manual [5], celiac axis nodes and paraesophageal nodes in the neck are included as regional lymph nodes, whereas supraclavicular lymph nodes (SCLNs) are defined as distant lymph nodes. Thus, patients with SCLN metastasis should be classified as having stage IV disease and consequently excluded from curative surgery [6, 7].

In previous decades, two-field lymphadenectomy involving the abdomen and mediastinum has remained the mainstay treatment for resectable esophageal cancer [8–12]. However, since SCLNs are not included in two-field lymphadenectomy, SCLNs could be investigated only in patients received three-field lymphadenectomy. Three-field lymphadenectomy has long been performed in some Asian countries, including China [13–17]. Several studies have reported that the use of three-field lymphadenectomy would help to achieve long term survival, even in cases of SCLN metastasis [16–20]. The adverse effect of SCLN metastasis appears to be less than that of visceral metastasis. Notably, as the population having the highest risk of esophageal cancer [1], Asian patients constitute only 25.2% (1,168 of 4,627 patients) of the database used to elaborate the 7^th^ AJCC staging system of esophageal cancer [21, 22]. On these basis, we believe that more data from Asian patients are essential for determining the role of SCLN metastasis in esophageal cancer.

Therefore, in this study, we evaluated a single-institution database collected in southern China over a long period. The aim of this study is to elucidate the effect of SCLN metastasis on long-term survival and to clarify the role of SCLN in established esophageal squamous cell carcinoma (ESCC).

## RESULTS

### Association between SCLN status and clinicopathological characteristics

A total of 1156 patients were enrolled as the target population. The median number of metastatic lymph nodes was two, ranging from one to 13. SCLN metastasis was found in 183 (15.8%) patients. We found that SCLN metastasis was independent of age, gender, pathological T status, and tumor cell differentiation. In the SCLN metastasis group, the proportion of patients with metastasis involving seven or more nodes was higher than that of patients without SCLN metastasis (16.4% *vs*. 3.2%); whereas the proportion of patients with 1-2 LNM was lower than that of patients without SCLN metastasis (31.7% *vs*. 65.5%). Regarding tumor location, SCLN metastasis was more common in the upper esophagus (22.4%, 43/192) and was less common in the lower esophagus (10.8%, 11/102) (*P* = 0.013). Since a previous study has indicated recurrent laryngeal nerve node is a marker of SCLN [16], we also analyzed their association. In this study, the rate of recurrent laryngeal nerve node metastasis, which was only 15.0% (146/973) in patients without SCLN metastasis, increased to 24.0% (44/183) in patients with SCLN metastasis (*P* = 0.002). Associations that were found between SCLC status and clinicopathological parameters are shown in Table 1.

**Table 1:** Association between SCLN status and clinicopathological parameters

		SCLN status		
Characteristic	Total	Negative	Positive	*P* value*
Age	1156	973	183	
≤60	790 (68.3)	663 (68.1)	127 (69.4)	0.737
>60	366 (31.7)	310 (31.9)	56 (30.6)	
Gender				
Male	898 (77.7)	763 (78.4)	135 (73.8)	0.166
Female	258 (22.3)	210 (21.6)	48 (26.2)	
Tumor location				
Upper	192 (16.6)	149 (15.3)	43 (23.5)	0.013
Middle	862 (74.6)	733 (75.3)	129 (70.5)	
Lower	102 (8.8)	91 (9.4)	11 (6.0)	
Pathological T status				
T1	39 (3.4)	34 (3.5)	5 (2.7)	0.647
T2	179 (15.5)	150 (15.4)	29 (15.8)	
T3	709 (61.3)	602 (61.9)	107 (58.5)	
T4a	229 (19.8)	187 (19.2)	42 (23.0)	
Tumor cell differentiation				
Good	200 (17.3)	164 (16.9)	36 (19.7)	0.357
Moderate	817 (70.7)	687 (70.6)	130 (71.0)	
Poor	139 (12.0)	122 (12.5)	17 (9.3)	
Number of positive lymph nodes				
1-2	696 (60.2)	638 (65.6)	58 (31.7)	< 0.001
3-6	399 (34.5)	304 (31.2)	95 (51.9)	
7-	61 (5.3)	31 (3.2)	30 (16.4)	
Adjuvant treatment				
None	658 (56.9)	555 (57.0)	103 (56.3)	0.329
Chemotherapy	219 (18.9)	191 (19.6)	28 (15.3)	
Radiotherapy	153 (13.2)	126 (12.9)	27 (14.8)	
Chemoradiotherapy	126 (10.9)	101 (10.4)	25 (13.7)	
Recurrent laryngeal nerve node status				
Negative	966 (83.6)	827 (85.0)	139 (76.0)	0.002
Positive	190 (16.4)	146 (15.0)	44 (24.0)	

* Pearson *χ*^2^ value.

SCLN, supraclavicular lymph node.

### Does SCLN indicate distant or regional metastasis for thoracic ESCC?

When SCLNs were classified as distant nodes (the current classification), 638 patients (55.2%) were diagnosed as having pathological N1 status, 304 patients (26.3%) were considered N2, 31 (2.7%) were considered N3, and 183 patients (15.8%) were considered M1; thus, 149 patients (12.9%) were diagnosed as having a pathological IIB status, 423 patients (36.6%) were considered IIIA, 197 patients (17.0) were considered IIIB, 204 patients (17.6%) were considered IIIC, and 183 patients (15.8%) were considered IV.

When the SCLNs were classified as regional nodes (the modified classification), 696 patients (60.2%) were diagnosed as having pathological N1 status, 399 patients (34.5%) were considered N2, and 61 (5.3%) were considered N3; thus, 166 patients (14.4%) were diagnosed as having pathological IIB status, 470 patients (40.7%) were considered IIIA, 254 patients (22.0%) were considered IIIB, and 266 patients (23.0%) were considered IIIC.

At the last follow-up checkpoint on June 30, 2016, 615 patients (53.2%) remained alive. The median survival time was 38.0 months. The 1-year, 3-year, and 5-year OS values were 84.0%, 51.5%, and 43.5%, respectively.

**Figure 1:**
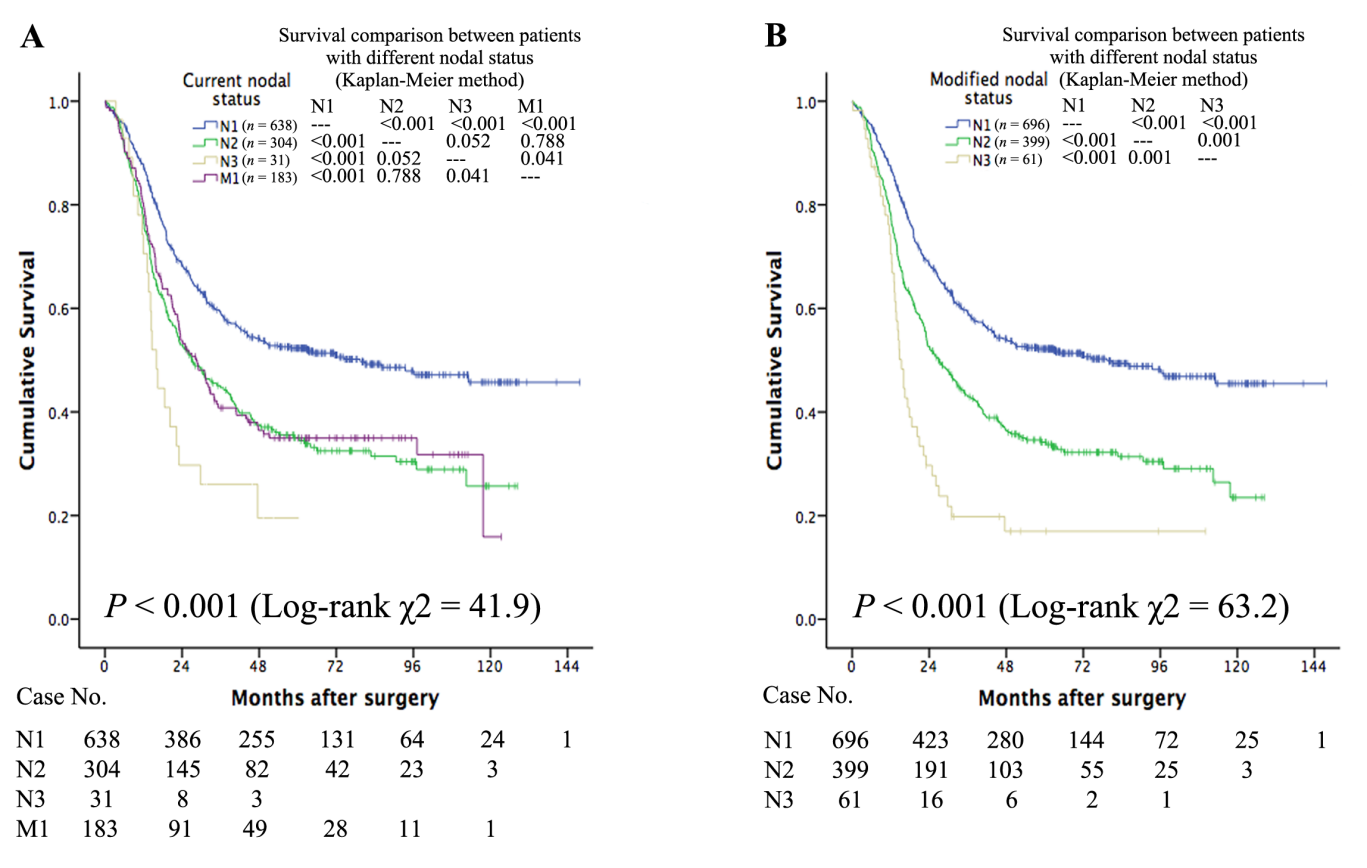
When SCLNs are considered distant nodes, the long-term survival of patients with M1 status is similar to that of patients with N2 status (*P* = 0.788) but is better than that of patients with N3 status (*P* = 0.041) (**Figure 1A**). When SCLNs were considered regional nodes, the three Kaplan-Meier survival curves did not overlap each other (**Figure 1B**).

**Figure 2:**
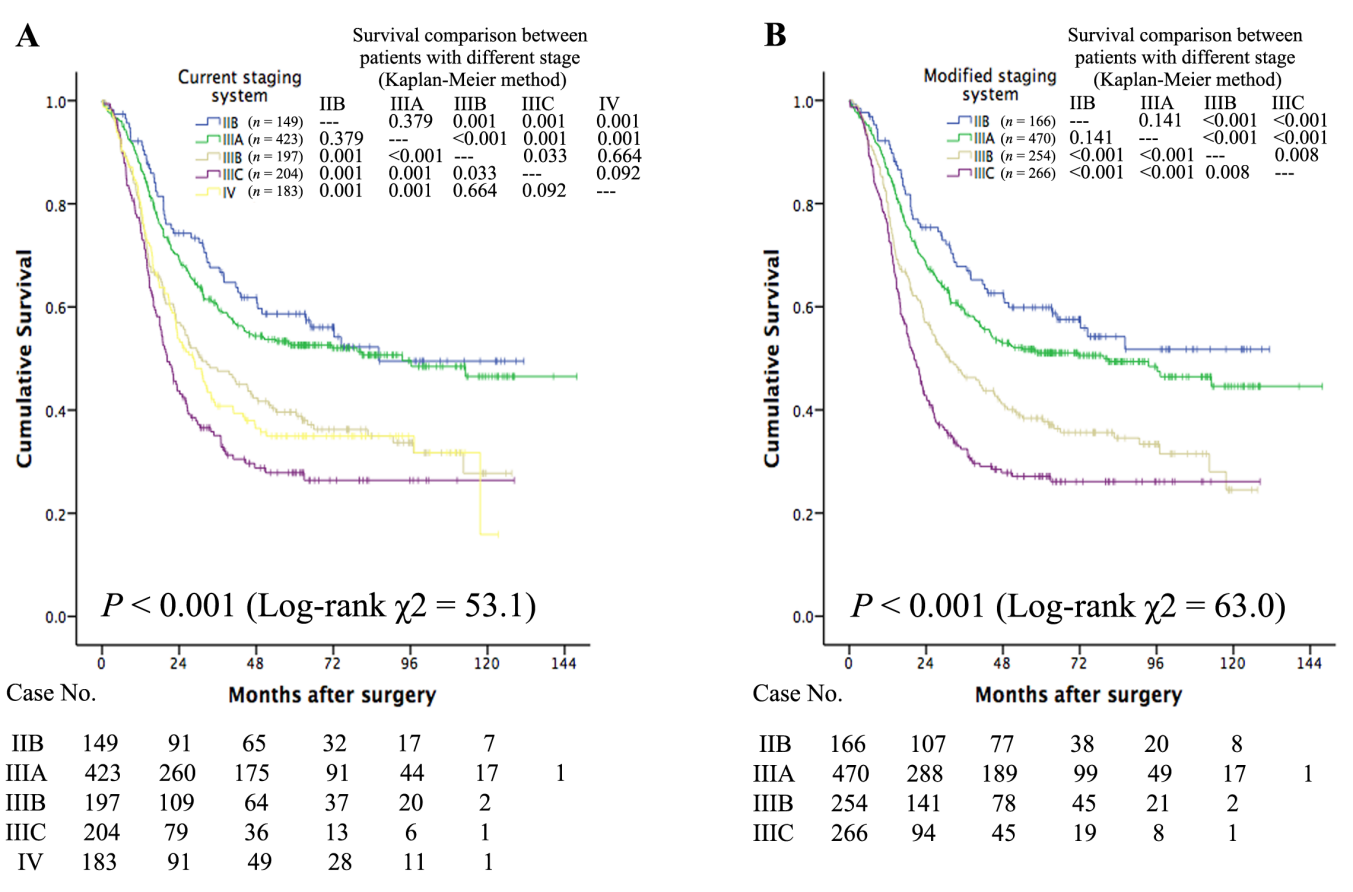
When SCLNs were considered distant nodes, the long-term survival of stage IV patients is similar to that of patients at stage IIIB (*P* = 0.664) but better than that of patients at stage IIIC (*P* = 0.092) (**Figure 2A**). When SCLNs were considered regional nodes, the prognosis was well stratified by the four Kaplan-Meier survival curves (**Figure 2B**).

As shown in Figure 1, patients with different nodal status present different survival values. When SCLNs were considered as distant nodes, the long-term survival of cases with M1 status was similar to that of cases with N2 status (*P* = 0.788) but better than that of cases with N3 status (*P* = 0.041) (Figure 1A). When SCLNs were considered as regional nodes, the three Kaplan-Meier survival curves did not overlap (Figure 1B). Figure 2 depicts the survival curves for the different stages. When SCLNs were considered as distant nodes, the long-term survival of stage IV cases was similar that of stage IIIB cases (*P* = 0.664) but better than of stage IIIC cases (*P* = 0.092) (Figure 2A). When SCLNs were considered as regional nodes, the prognosis was well stratified by the four Kaplan-Meier survival curves (Figure 2B).

The results of the univariate survival analyses are listed in Table 2. Age (*P* = 0.009), gender (*P* = 0.001), pathological T status (*P* < 0.001), current nodal status (*P* < 0.001), and modified nodal status (*P* < 0.001) were significantly associated with OS.

**Table 2:** Univariate survival analysis

Characteristic	5-year survival rate (%)	Log-rank *χ*^2^ value	*P* value
Total			
Age		6.742	0.009
≤60	46.2		
>60	38.5		
Gender		10.712	0.001
Male	41.0		
Female	53.4		
Tumor location		0.261	0.878
Upper	43.4		
Middle	42.4		
Lower	46.0		
Pathological T status		31.333	< 0.001
T1	67.7		
T2	50.1		
T3	45.7		
T4a	29.6		
Tumor cell differentiation		3.312	0.193
Well	45.4		
Moderate	42.1		
Poor	49.3		
Adjuvant treatment		4.256	0.235
None	46.2		
Chemotherapy	39.3		
Radiotherapy	43.2		
Chemoradiotherapy	37.1		
Current staging system (considering SCLNs as distant metastasis)			
Nodal status		41.901	< 0.001
N1	52.0		
N2	35.8		
N3	19.2		
M1 (SCLC metastasis)	34.6		
Modified staging system (considering SCLNs as regional metastasis)			
Nodal status		63.213	<0.001
N1	52.0		
N2	34.4		
N3	17.0		

SCLN, supraclavicular lymph node.

Since both the current nodal status and the modified nodal status were found as prognostic factors in the univariate analyses, two separate multivariate models were developed: one included age, gender, pathological T status, and current nodal status; and the other included age, gender, pathological T status, and modified nodal status. As shown in Table 3, using the current model in accordance with the current staging system, age (HR = 1.319), gender (HR = 0.723), pathological T status (HR = 1.372), and current nodal status (HR = 1.110) were found as independent prognostic parameters for OS. Additionally, using the modified model in accordance with the modified staging system, age (HR = 1.352), gender (HR = 0.765), pathological T status (HR = 1.287), and current nodal status (HR = 1.588) were found as independent prognostic parameters for OS. The performance of the current and modified systems were then quantified based on the likelihood ratio chi-square, AIC, and c-index. As shown in Table 4, the AIC value for the modified version was smaller than that for the current version, indicating that the modified version yields a better prognostic stratification; the c-index value was larger for the modified version than for the current version (*P* = 0.0092), indicating that it is more informative regarding patient outcome.

**Table 3:** Multivariate cox regression analysis

Factor	HR (95% CI)	*P* value
Current model		
Age (≤60/>60)	1.319 (1.104-1.576)	0.002
Gender (Male/Female)	0.723 (0.581-0.900)	0.004
Pathological T status (T1/T2/T3/T4a)	1.372 (1.202-1.566)	< 0.001
Current nodal status (N1/N2/N3/M1)	1.110 (1.052-1.172)	< 0.001
Modified model		
Age (≤60/>60)	1.352 (1.131-1.616)	0.001
Gender (Male/Female)	0.765 (0.614-0.954)	0.018
Pathological T status (T1/T2/T3/T4a)	1.287 (1.124-1.474)	< 0.001
Modified nodal status (N1/N2/N3)	1.588 (1.383-1.822)	< 0.001

SCLN, supraclavicular lymph node; HR, hazard ratio; CI, confidence interval.

**Table 4:** Comparison between two multivariate cox regression models

Cox model	-2 log likelihood	AIC value	C-index (95% CI)	*P* value
Current model	6949.764	6957.8	0.6069 (0.5815-0.6323)	0.0092
Modified model	6864.936	6872.9	0.6242 (0.5989-0.6494)	

Current model: considering SCLNs as distant nodes; Modified model, considering SCLNs as regional nodes; AIC value, Akaike information criterion value; C-index, concordance index; CI, confidence interval.

## DISCUSSION

Esophageal cancer with SCLN metastasis is generally regarded as a systemic disease and is commonly excluded from indications for curative surgery [6, 7]. However, in some Asian countries, especially Japan, three-field lymphadenectomy with cervical lymph node dissection including SCLN is aggressively performed [13–20]. As reported in several studies, patients with SCLN metastasis commonly present good outcomes, unlike stage IV disease; therefore, considering SCLN metastasis as distant metastasis may be biased [16, 17, 19, 20]. In this study, we first plotted survival curves and found that the current system did not stratify outcome well in patients with N2, N3, and M1 status. Then, using a multivariate Cox regression analysis, the modified system was quantified as having a smaller AIC value but a larger c-index, indicating that it is more informative regarding patient outcome. Therefore, our results suggest considering SCLNs as regional lymph nodes in thoracic ESCC. Some recent studies have reported similar results [17, 19].

Based on previous studies from Japan, the 5-year survival of patients with SCLN metastasis commonly ranges from 24.1% to 28.7% [15, 16, 19]. However, this value was increased to 34.6% in this study. We would like to attribute this discrepancy to the variance of metastatic node number. In this study, the median number of involved nodes was four in the SCLN metastasis cohort. For comparison, in the aforementioned three studies, the median number of involved LNs for patients with SCLN metastasis was six in one study [19] and unclear in the other two studies [15, 16]. As indicated by many studies, the outcomes of ESCC are not simply impacted by SCLN status but are primarily determined by the number of involved nodes including SCLNs; survival is commonly found to be worse in patients with SCLN metastasis, not simply because of the SCLN metastasis, but because of the number of involved nodes [13, 15, 19, 23]. A recent study found similar results to those obtained in our study. In that study, among patients with SCLN metastasis, the 5-year survival rate was 42.3%, 40.5%, and 30.0% for upper, middle, and lower esophageal cancer, respectively [18].

In the previous 6^th^ AJCC staging classification, both SCLN and celiac axis nodes were defined as distant metastasis [24]. During the past two decades, along with an increase in distal esophageal cancer and gastroesophageal junction carcinoma in Western countries, cases with celiac axis metastasis have increased. Better survival of patients with celiac node metastasis has also been reported. As a result, celiac axis nodes have been classified as regional lymph nodes by the Worldwide Esophageal Cancer Collaboration in the 7^th^ AJCC staging classification [5]. Cases from Western countries constitute more than 70% of the database used to elaborate the 7^th^ AJCC staging system, and the most pathological type was found to be adenocarcinoma [21, 22]. In Asian patients, however, squamous cell carcinoma remains the predominant histological cell type of esophageal cancer [1, 10, 14]. Furthermore, SCLNs could be investigated only in patients who received three-field lymphadenectomy. Although this procedure has been widely applied in Asian countries for a long time, it is barely applied in Western countries. Therefore, the current staging system may be biased regarding SCLN, especially in the ESCC cohort. Based on a large sample of data obtained in southern China, our study supplements the strong evidence supporting SCLNs as regional nodes in ESCC. Together with recent studies in Japanese populations [16, 17, 19], our study should be as valuable for the revision of staging as the Worldwide Esophageal Cancer Collaboration data.

Theoretically, since SCLNs are regional lymph nodes, additional SCLN dissection should be performed for every patient with thoracic ESCC to determine more accurate staging and obtain longer survival. However, aggressive extended lymphadenectomy commonly leads to significantly enhanced perioperative morbidity and mortality [9]. Thus, in recent decades, increasingly enthusiastic debate about the necessity of three-field lymphadenectomy has occurred. To date, no clear consensus has been reached. To assess the feasibility of selective SCLN dissection, we investigated the association between SCLN status and clinicopathological parameters. Based on our results, the frequency of SCLN metastasis is highest (22.4%, 43/192) in upper esophageal cancer. A similar tendency has been observed in previous studies [14–16, 19]. In addition, a recent study indicated that recurrent laryngeal nerve node metastasis may increase the risk of SCLN metastasis [16]. This phenomenon was also observed in our study. For these reasons, we propose that three-field lymphadenectomy be routinely performed on patients with upper thoracic ESCC. For patients with middle or lower thoracic ESCC, intraoperative frozen section of recurrent laryngeal nerve nodes should be carried out; additional cervical dissection should be performed for those with positive recurrent laryngeal nerve nodes.

These findings should be considered in the context of certain limitations in our study. First, the study was retrospective in nature. Thus, we are participating in a multi-center prospective randomized study that funded by the National Science and Technology Support Plan (Project No., 2015BAI12B00) to verify our results. Second, heterogeneities, including those associated with patient characteristics or the determination of treatment, were unavoidable due to the lengthy study period, although we conducted subgroup and multivariate analyses to minimize these confounders. However, we believe that this nonselective and nonmatching population results in a more generalized significance of this study. Furthermore, we recruited patients from the database of a single institution, which has carried out three-field lymphadenectomy for a long period in a mature fashion; this consistency and use of a standard SCLN dissection procedure ensured the quality and reliability of our results. Third, the sample size of our SCLN metastasis cohort was small (*n* = 183), hindering a further subgroup analysis. For example, traditional opinion holds that tumor cells generally attack proximal nodes at the start of metastasis; distal node metastasis commonly cause more harm on prognosis than proximal node metastasis. According to this hypothesis, the impact of SCLN metastasis on long-term survival may be more prominent in patients with lower thoracic ESCC. Thus, we conducted a directed subgroup analysis, which showed that the outcome was similar between patients with and without SCLN metastasis in this cohort (*P* = 0.240). However, due to the limited sample size of the SCLN metastasis group (*n* = 11), this result is greatly biased. Therefore, a further prospective multicenter study is warranted.

In conclusion, SCLNs should be considered as regional lymph nodes in thoracic ESCC to obtain a better stratification of overall survival.

## PATIENTS AND METHODS

This study was approved by the Medical Ethics Committee of Fujian Provincial Cancer Hospital. Informed written consent was obtained from all participants. Patients who were diagnosed with ESCC and underwent transthoracic esophagectomy and three-field lymphadenectomy at the Thoracic Surgery Department of Fujian Provincial Cancer Hospital from January 1999 to December 2008 were screened for study recruitment.

All patients with pathologically confirmed ESCC who fit the following inclusion criteria were included in the analysis: (1) received transthoracic esophagectomy and three-field lymphadenectomy; (2) pathological T status of T1, T2, T3, or T4a; (3) pathological lymph nodal metastasis (including SCLN metastasis); (3) without visceral metastasis; (4) microscopically complete resection (R0); (5) resection was not preceded by chemotherapy, radiotherapy, or other anti-cancer treatment; and (6) the records contained complete basic clinicopathological information. We did not include patients who received neoadjuvant treatment because this treatment may alter SCLN status [25]. Patients with common hepatic or splenic artery node metastasis were also excluded.

Analysis of clinical stage was performed using barium esophagography, computed tomography scanning (involving the chest, abdomen and cervical region), and electronic and ultrasound gastroscopy. PET/CT was not routinely carried out.

### Surgical procedure

The surgical procedure for total esophagectomy and three-field lymphadenectomy has been described previously [15]. Generally, we carried out an initial right-sided thoracotomy for esophagectomy and mediastinum lymphadenectomy, followed by a midline laparotomy for the mobilization of the substituent (mostly stomach) and abdominal lymphadenectomy and eventually a U-shape cervicotomy for anastomosis and bilateral cervical lymphadenectomy.

### Tumor classification

Pathologic staging was reassessed based on the 7^th^ AJCC staging system [5]. Lymphatic nodes were named according to the guideline of the Japanese Society for Esophageal Diseases (JSED), as mentioned in a previous study [14]. The dissected cervical lymph nodes included SCLNs and cervical paraesophageal nodes. Paraesophageal nodes, recurrent laryngeal nerve nodes, paratracheal nodes, subcarinal nodes, hilar nodes, posterior mediastinal nodes, and diaphragmatic nodes were dissected during thoracotomy. Abdominal lymph nodes that were dissected during the routine procedures involved the paracardial nodes and nodes around the left gastric artery and the celiac axis.

### Adjuvant treatment

In our hospital, we suggested adjuvant treatment for ESCC with LN metastasis. Treatment options were selected based on tumor stage, doctor opinion, patient physical status, and patient desire. Generally, adjuvant treatment was started at 4-8 weeks after operation. Chemotherapy was typically applied as a platinum-based, two-drug regimen for 4-6 cycles. Postoperative radiotherapy was delivered to the anastomosis, supraclavicular, and mediastinal lymphatics, with a total dose of 45-60 Gy.

### Follow-up

After primary treatment, most patients were followed up in an outpatient clinic every three months for the first two years, every six months for years 3-5, and every 12 months thereafter. For patients who could not afford regular follow-up visits, a telephone follow-up was performed instead. Regular assessment included physical examination, blood test, endoscopy, chest X-ray, and ultrasound test. Computed tomography scanning of the chest, abdomen, and cervical region was performed at least once each year. Survival status was re-verified using the best available method in June 2016. The median time from the date of surgery to the last censoring was 57.8 months.

### Statistical analysis

Statistical analysis was performed using the SPSS 22.0 software package (SPSS Inc., Chicago, IL). Statistical significance was assumed at a two-sided probability value of < 0.05. Correlations between SCLN status and clinicopathological characteristics were assessed using Pearson's *χ*^2^ test. Overall survival (OS) was defined from the date of surgery to the date of death or final follow-up. Censored cases were defined as patients who were lost during follow-up or remained alive at the end. Survival rate was calculated using the Kaplan-Meier method, and differences between the curves were assessed using the log-rank test. Factors that proved to have statistical significance (*P* < 0.1) in univariate survival analyses were introduced into multivariate analyses. Multivariate analysis was performed using the Cox proportional hazard model using the backward logistic regression stepwise procedure for variable selection. To illuminate the role of SCLNs, SCLNs were classified not only as distant LNs based on the current staging system but also as regional LNs based on the modified staging system. To measure the homogeneity of direct comparisons of the two different versions of the staging system, the likelihood ratio *χ*^2^ test, which is related to the Cox regression model, was used. The discriminatory ability and monotonicity of gradient assessments were measured using the linear trend *χ*^2^ test of survival curves according to the classification of the two different staging systems. The Akaike information criterion (AIC) and the concordance index (c-index) was applied to the Cox proportional hazard regression model to correct for potential bias in comparing prognostic systems with different numbers of stages. AIC was defined as follows: AIC= -2 log maximum likelihood + 2 × (the number of parameters in the model). Smaller AIC values indicate better goodness-of-fit [26]. The C-index was calculated using R software (version 3.3.2), and larger c-index values indicate better predicted precision of outcome [27].
